# Protective effects of Shenkang injection against diabetic kidney disease via p38 MAPK/NFκB/MCP-1/CCR2 pathway inhibition

**DOI:** 10.3389/fendo.2025.1596000

**Published:** 2025-09-09

**Authors:** Xin Zhou, Sitong Wang, Ge Jin, Kaidong Zhou, Yanmo Cai, Zongjiang Zhao

**Affiliations:** School of Traditional Chinese Medicine, Beijing University of Chinese Medicine, Beijing, China

**Keywords:** Shenkang injection, diabetic kidney disease, p38 MAPK, NFkapapB, CCR2, MCP-1

## Abstract

**Background:**

Diabetic kidney disease (DKD) is a complication of microvascular disease that occurs in the late stages of diabetes. Shenkang injection (SKI) has shown promising effects on DKD, but its mechanism has not been fully elucidated. Therefore, this study aims to investigate the mechanism by which SKI reduces kidney inflammatory injury and delays DKD progression.

**Methods:**

Several db/m mice were used as the control group, while db/db mice were randomly divided into the model group, the dagliflozin group, and the SKI group. HK-2 cells were cultured *in vitro* and divided into the control group, high glucose group, SKI group, and SB203580 group. In this study, the therapeutic effect of SKI on DKD was evaluated by observing the general condition of the mice alongside blood and urine biochemical indices. TEM, HE staining, PAS staining, and Mallory staining were utilized to assess the pathological injury of renal tissue. Immunohistochemistry, WB, and real-time qPCR were employed to detect the expression of the key proteins involved in the mechanisms in mouse renal tissue and HK-2 cells.

**Results:**

The results indicated that the general condition and kidney injury were significantly improved in the SKI group, as evidenced by reduced urinary protein quantification, urinary albumin-to-creatinine ratio, SCr, and urea levels (P<0.01). Routine staining and TEM analyses demonstrated significant improvement in podocyte injury and renal interstitial fibrosis. The CCK-8 results demonstrated high cell survival rates in the SKI group. There were significant decreases in p-p38, p-NFκB, MCP-1, and CCR2 levels (P<0.05, P<0.01), with no statistical differences observed for p38 and NFκB. Real-time qPCR revealed significant reductions in MCP-1 and CCR2 mRNA expression in the SKI group (P<0.01).

**Conclusion:**

SKI can reduce kidney inflammatory damage in db/db mice, improve kidney function, and delay the progression of diabetes. The mechanism may involve the reduction of MCP-1/CCR2 activation through the p38 MAPK/NFκB signaling pathway.

## Introduction

Diabetes mellitus (DM) is a chronic disease characterized by hyperglycemia. According to epidemiological studies, the number of people with diabetes mellitus rose to 463 million worldwide by 2019 and is predicted to increase to 578 million in 2030 and 700 million in 2045 (1). Diabetic kidney disease (DKD) is a complication of microvascular disease that typically occurs in the late stage of diabetes, with an incidence rate of approximately 40% among diabetic patients (2).

DKD is primarily characterized by a decrease in glomerular filtration rate accompanied by persistent proteinuria (3, 4). Although DKD remains the leading cause of end-stage renal disease (ESRD) and can ultimately result in the need for dialysis, its prevalence has not decreased; rather, it has increased annually over the past three decades (5). Currently, Western approaches to diagnosing and treating DKD mainly focus on lowering blood pressure, managing glucose levels, regulating lipid levels, reducing inflammation, and decreasing proteinuria. However, these measures can only delay the progression of early DKD and do not effectively reverse the disease (6).

With the advancement and promotion of traditional Chinese medicine (TCM) research, its clinical applications are becoming increasingly widespread. Several studies have demonstrated that due to the safety and low side effects of TCM, various TCM preparations have been widely utilized in treating DKD (7, 8). Among these, Shenkang injection (SKI) stands out as it is primarily composed of four types of traditional Chinese medicine: rhubarb, *Astragalus*, *Salvia miltiorrhiza*, and safflower. It is known for its effects in reducing stasis and relieving turbidity, invigorating qi and blood circulation, and supporting overall health and vitality. SKI is used not only in patients with renal failure but also in those with lung infections, acute exacerbations of chronic obstructive pulmonary disease, heart failure, malignancy, diabetes mellitus, hyperuricemia, and other diseases (9).

Hyperglycemia causes cellular damage and triggers the release of pro-inflammatory mediators, including the chemokines MCP-1 and TNF-α, which promote macrophage infiltration in the kidney, leading to inflammation and proteinuria (10). MCP-1 promotes macrophage infiltration. Previous studies have demonstrated overexpression of MCP-1 in the kidneys of animal models with DKD. MCP-1 contributes to renal injury not only by inducing monocyte recruitment but also through direct activation of resident renal cells (11). CC chemokine receptor 2 (CCR2), the receptor for MCP-1, is also involved in these pathological processes (12). To counteract these detrimental effects, studies have shown that SKI alleviates glomerulosclerosis and interstitial fibrosis (13). Moreover, when combined with alprostadil injection, SKI can significantly improve renal function and reduce inflammation in DKD. Additionally, SKI can regulate the production of various cytokines, inhibit inflammation, and delay the progression of DKD (14). Moreover, it has demonstrated good safety and effectiveness in the clinical treatment of renal diseases such as DKD and chronic renal failure (15, 16). Furthermore, pharmacological studies have found that SKI can reduce proteinuria by effectively alleviating anemia and improving kidney microcirculation, thereby inhibiting pathological kidney proliferation, which helps reduce kidney damage, promote the repair of damaged tissues, and ultimately improve kidney function (17). Finally, animal experiments indicate that SKI can downregulate the activity of NK cells through the STING/TBK1/IRF3 signaling pathway, resulting in reduced renal fibrosis (13).

The mitogen-activated protein kinase (MAPK) signaling pathway represents a tertiary kinase system that jointly regulates cell growth, differentiation, stress, and various inflammatory responses through multi-stage regulation and continuous amplification of three kinases. Among its branches, the p38 MAPK signaling pathway plays a crucial role in promoting the production of inflammatory cytokines and oxidative stress (18). At the center of this pathway is p38, a serine kinase that serves as a key mediator in multiple pro-inflammatory responses. Importantly, inhibitors of p38 can alleviate various inflammatory diseases (19). Supporting this, one study found that the single blockade of p38 MAPK signaling relieved renal fibrosis in a mouse model of unilateral ureteric obstruction (20).

In addition to the MAPK pathway, the nuclear factor NFκB is another critical player in inflammation. As a nuclear transcription factor, NFκB triggers the expression of pro-inflammatory genes, thereby regulating inflammation and immune responses within cells (21). Together, the MAPK and NFκB signaling pathways are recognized as core mechanisms underlying the regulation of inflammatory mediators and cytokines. The p38 MAPK pathway can be activated by various stimuli, including high glucose, ultraviolet radiation, and inflammatory cytokines (22). Once activated, it regulates mRNA translation through nuclear transcription factors such as NF-κB, leading to the production of pro-inflammatory mediators (23), while also influencing cellular processes such as proliferation and differentiation. The activation of pro-inflammatory factors can modulate the expression and function of MCP-1. MCP-1 plays a central role in inflammatory responses, with CCR2 being its primary receptor. Upon MCP-1 binding to CCR2, the MCP-1/CCR2 axis is activated (24). This activation promotes the chemotaxis and activation of inflammatory cells (25). Therefore, we believe that SKI may alleviate the inflammatory response caused by DKD and reduce renal injury through the p38 MAPK and NFκB signaling pathways.

## Materials and methods

### Experimental verification

#### *In vivo* studies

##### Laboratory animals

Thirty SPF male db/db mice, 7 weeks of age, with a body weight of (39.34 ± 0.34) g, and ten SPF male db/m mice, also 7 weeks of age, with a body weight of (28.80 ± 0.46) g, were purchased from Changzhou Carvens Experimental Animal Co., Ltd., Animal Certificate No. 202247475, License No. SCXK (Su) 2021-0013. The experimental mice were kept in the animal room of the Beijing University of Chinese Medicine at a room temperature of 24–26°C, relative humidity of 50–60%, and had free access to drinking water.

##### Main reagents

SKI (202, 107, 108) was provided by Xi’an Century Shengkang Pharmaceutical Co., Ltd. (Xi’an, China). Other reagents included: dagliflozin (NH3205); sodium pentobarbital (Beijing Chemical Reagent Company, catalog number: 020402); electron microscope fixative (Servicebio, catalog number: G1102); primary antibodies for immunohistochemistry and western blotting (WB): p-p38 (ImmunoWay, catalog number: YP0338), p38 (CST, catalog number: # 4511), MCP-1 (Thermo Fisher, catalog number: MA5-17040), CC motif chemokine receptor 2 (CCR2) (Proteintech, catalog number: 16153-1-AP), NFκB (CST, catalog number: 8242S), p-NFκB (ABways, catalog number: F107172), β-actin (CST, catalog number: # 4970); secondary antibodies: goat anti-rabbit (Abcam, catalog number: ab672), goat anti-rat (Abcam, catalog number: ab67891); hematoxylin (Solaibao, catalog number: G1080); eosin (Solaibao, catalog number: G1100); a Mallory Three-color Staining Kit (Solaibao, catalog number: G1355); a Glycogen Staining Solution (Beijing Legen, catalog number: DG0005); RIPA tissue/cell lysate (Lablead, catalog number: R1091); a BCA Protein Quantification Kit (Lablead, catalog number: B5001); a Protease Inhibitor Cocktail (Lablead, catalog number: C0101); and a Phosphatase Inhibitor Cocktail (Lablead, catalog number: R100).

##### Establishment and grouping of mice

All animals were fed for 5 weeks and had ad libitum access to food. Blood glucose was collected from db/db mice, with blood glucose values of 11.1 mmol/L and urinary albumin-to-creatinine ratio (UACR) >3 mg/mmol serving as the entry criteria (26, 27). Mice were randomly stratified by weight into the model group, dagliflozin group, and SKI group, with ten mice in each group. The db/m mice served as the normal group. Based on body surface area, dagliflozin (1.6 mg/kg) and SKI (15.6 mL/kg) were administered, while the model and normal groups received 0.1 mL per 10 g of body mass, once daily for 12 weeks.

#### Observing indicators

##### General status

The mental state, fur condition, limb movement, activity levels, food, and water intake of each group were observed weekly, and weight measurements were recorded every 3 weeks.

##### Quantitative determination of urine protein at 24 hours

Urine from each group was collected for 24 hours before administration and at week 12 of dosing. Urine protein from each group of mice was extracted using a liquid protein extraction kit, with concentration determined by BCA. The 24-hour urinary protein quantification was calculated. Urinary albumin and creatinine levels were measured using an automated biochemical analyzer, and UACRs were also calculated.

##### Fasting blood glucose measurement

Blood glucose levels were measured from the tail tip before fasting and at week 12 after fasting for 8 hours with water.

##### Detection of blood biochemical indicators

Serum biochemical indicators were measured, including urea, serum creatinine (SCr), total cholesterol (TC), triglycerides (TG), and low-density lipoprotein (LDL).

##### Renal pathology analysis

Twelve weeks after drug administration, the mice were anesthetized intraperitoneally with a 1% pentobarbital solution. Renal tissue was prepared through a longitudinal cut, routinely fixed, paraffin-embedded, sectioned, and stained using hematoxylin and eosin (HE), Mallory stain, and a glycogen stain. Additionally, a 1 mm³ sample of renal tissue was obtained from each group and fixed in a solution of 2.5% glutaraldehyde and osmium tetroxide.

##### Immunohistochemical analysis

Paraffin sections were retrieved from 4°C and dewaxed using routine methods. They were treated with 0.01% Triton and incubated at 37°C for 10 minutes. Hydrogen peroxide was added to methanol for 10 minutes, followed by antigen retrieval for 15 minutes after repeated washes with PBS. Goat serum was used for blocking for 1 hour. The primary antibodies p38 (1:100), phospho-p38 (1:500), NFκB (1:400), phospho-NFκB (1:200), MCP-1 (1:200), and CCR2 (1:200) were applied and incubated at 4°C overnight. After cleaning with PBS, the corresponding secondary antibody was added and incubated at 37°C for 30 minutes. DAB was used for coloration, followed by hematoxylin nuclear staining. After gradient dehydration and transparency treatments, samples were sealed with neutral resin. Photographs were taken under an optical microscope, and image analysis was conducted with Image J software. Buffy particles indicated positive expression, and six random fields were selected for each group.

##### Western blot

Approximately 100 mg of frozen mouse kidney tissue was removed from the 80°C freezer, cut into small pieces, and washed with saline. Lysate homogenate was added, and tissues were lysed on ice. Samples were centrifuged at a low temperature and high speed to obtain the supernatant. Protein concentration was determined using the BCA assay, with sample buffer added and heated in a metal bath for 10 minutes. A 10% SDS-PAGE gel was prepared, and a sample of 30 µg protein was loaded for gel electrophoresis. A constant voltage was applied for transfer. Subsequently, a 5% non-fat milk powder solution was used to block the membrane at room temperature for 2 hours. After washing with TBST, primary antibodies β-actin (1:1000), p38 (1:2000), phospho-p38 (1:5000), NFκB (1:2000), phospho-NFκB (1:2000), CCR2 (1:1000), and MCP-1 (1:2000) were added. Following further TBST washes, the corresponding secondary antibody (1:2000) was incubated for 2 hours at room temperature. ECL was used, and exposure imaging was performed. Image J was utilized for image analysis, and the ratio of the grayscale of the internal reference band was used to determine the relative expression level of the target protein.

##### Real time-qPCR

Mouse kidney tissue (100 mg) was cut on ice, and 1 mL of pre-cooled Trizol lysate was added. The tissue was electrohomogenized and left to stand on ice for 10 minutes. Chloroform was added, followed by centrifugation at 4°C, 15,000 r/min, for 15 minutes. The upper phase was transferred to isopropyl alcohol, centrifuged again, and the supernatant was discarded. The RNA pellet was washed with ethanol to precipitate, and 40 μl of DEPC water was added to dissolve the RNA. The RNA concentration and purity were measured. Reverse transcription was performed using a reverse transcription kit, following the manufacturer’s instructions. The reverse transcription conditions were as follows: 42°C for 15 minutes, 95°C for 5 minutes, 72°C for 5 minutes, and 4°C for cooling. After reverse transcription, amplification was carried out according to the reaction system: pre-denaturation at 95 °C for 2 minutes (1 cycle), thermal cycling at 95 °C for 15 seconds, and 60°C for 40 seconds (repeated for 40 cycles). The relative template content in each group was quantified using the CT value (P-value), normalized to the reference gene (β-actin), and calculated as 2^(-ΔΔCt). The primers were synthesized and purified by Biological Bioengineering (Shanghai) Co., Ltd (**Table 1**).

**Table 1:** Primer sequences.

Gene	Sequences (5’-3’)	bp
MCP-1	F: CACTCACCTGCTGCTACTCA	280
	R: TCAGATTTACGGGTCAACTTCAC	
CCR2	F: GTTACCTCAGTTCATCCA	117
	R: CAAGGCTCACCATCATCGTAGTC	
β-actin	F: TCCTGTGGCATCCACGAAACT	315
	R: GAAGCATTTGCGGTGGACGAT	

#### *In vitro* studies

##### Cell culture

HK2 cells (ATCC, lot CRL-2190TM) were cultured in DMEM/F12 media supplemented with 1% penicillin-streptomycin and 10% FBS and incubated at 37°C in a 5% CO_2_ atmosphere.

##### Main reagents

F12 DMEM sugar-free medium (Servicebio, catalog number: G4538); FBS (Servicebio, catalog number: G8003); penicillin-streptomycin mixture (Servicebio, catalog number: G4003); 0.25% trypsin (Servicebio, catalog number: G4013-100ML); D-(+)-glucose (Beyotime, catalog number: ST1228-250g); dimethyl sulfoxide (cell culture grade) (Solaibao, catalog number: D8371); laboratory-grade pure water (Servicebio, catalog number: G4701-500ML); 1×PBS (pH 7.4) (Servicebio, catalog number: G4202-500ML); SB203580 (Selleck, catalog number: S1076–25 mg).

##### Cell grouping and drug administration

The cells were divided into four groups: the normal group, high glucose group, SKI group, and SB203580 group. All groups, except for the normal group, were treated with high glucose medium (30 mmol/L glucose). The normal and high glucose groups were cultured with 8% FBS, while the SKI group was treated with 4% SKI injection (52) and 8% FBS, and the SB203580 group was treated with p38 inhibitors and 8% FBS. A concentration of SB203580 at 10 μmol/L was added 2 hours before administration for pre-stimulation. Cellular protein and mRNA were extracted 48 hours after each group’s intervention.

##### HK2 cell viability detection

HK2 cells in the log growth phase were seeded into 96-well plates at a density of 5000 cells per well and incubated at 37°C with 5% CO_2_. The cells were allowed to grow to 50% confluence, after which the medium was replaced with serum-free medium for 24 hours to synchronize the cells to the G0 phase. During this stage, the normal group, high sugar group, SKI group, and SB203580 (a p38 MAPK inhibitor) group were treated, and after 12, 24, 36, and 48 hours, CCK-8 solution was added to each well and incubated for an additional 2 hours. The absorbance was measured at 450 nm to calculate cell viability.

##### Western blot

HK-2 cells from each group were collected and homogenized with RIPA lysis buffer, then lysed on ice for 30 minutes. The supernatant protein was collected. Protein concentration was measured using the BCA assay. The sample buffer was added, and the samples were placed in a metal bath at 95°C for 10 minutes for protein denaturation. A 10% SDS-PAGE gel was prepared with 50 µg of protein loaded for gel electrophoresis. A constant voltage was applied for the electric transfer. A blocking solution of 5% non-fat milk powder was used to block the membrane at room temperature for 2 hours. Following the TBST washing period, primary antibodies—β-actin (1:1000), p38 (1:2000), phospho-p38 (1:5000), NFκB (1:2000), phospho-NFκB (1:2000), CCR2 (1:1000), and MCP-1 (1:2000)—were added and incubated at 4°C overnight. The corresponding secondary antibody was applied the next day and incubated at room temperature for 2 hours. ECL detection was performed using an exposure imaging system, and image analysis was carried out with ImageJ. The grayscale ratio of the target protein to the internal reference band was calculated as the relative expression level.

##### Real time-qPCR

HK-2 cells were collected, and 1 mL of pre-cooled Trizol lysis buffer was added. The samples were placed on ice for 10 minutes, followed by the addition of chloroform, and then centrifuged at 15,000 r/min for 15 minutes at 4°C. The upper liquid layer was collected, and an equal volume of isopropanol was added before being centrifuged again. The upper liquid was discarded, and the RNA precipitate was washed with ethanol. Finally, 40 µL of DEPC water was added to the mixture, thoroughly mixed, and the RNA concentration and purity were measured. The reverse transcription system was prepared according to the kit instructions, with the following reverse transcription conditions: 42°C for 15 minutes, 95°C for 5 minutes, 72°C for 5 minutes, and stored at 4°C indefinitely. After reverse transcription, amplification was performed according to the reaction system: pre-denaturation at 95°C for 2 minutes (1 cycle); thermal cycling at 95°C for 15 seconds and 60°C for 40 seconds, repeated for 40 cycles. The P-value corresponding to the CT value was used to quantify the relative content of templates in each group, normalized by the reference gene (β-actin), and calculated as 2^(-ΔΔCt). The primers were synthesized and purified by Biological Bioengineering (Shanghai) Co., Ltd (**Table 2**).

**Table 2:** Primer sequences.

Gene	Sequences (5’-3’)	bp
MCP-1	F: AAAGTCTCTGCCGCCCTTC	171
	R: CTTGCTGCTGGTGATTCTTCTAT	
CCR2	F: TGGCTGTGTTTGCTTCTGTC	230
	R: TCTCACTGCCCTATGCCTCT	
β-actin	F: CGGGACCTGACTGACTACC	292
	R: TGAAGGTAGTTTCGTGGATGC	

### Statistical methods

Statistical analysis was conducted using SPSS 26.0. Measurement data meeting a normal distribution were expressed as mean ± standard error of the mean (means ± SEM). One-way analysis of variance (one-way ANOVA) was used for comparisons, with equal variance tested by the LSD method and variances by Dunnett’s T3 method. A significance level of α=0.05 was set, with P<0.05 indicating statistically significant differences.

## Results

### Animal experiments

#### General state of the animals

Before administration, compared to the mice in the normal group, the mice in other groups exhibited signs of depression, loss of fur, slowed movement, reduced activity, and increased fat accumulation in the groin and axillary regions, along with significantly increased food, water, and urine volume. After administration, improvements were observed in the dapagliflozin and SKI groups.

#### Effect of SKI on body weight in db/db mice

**Figure 1:**
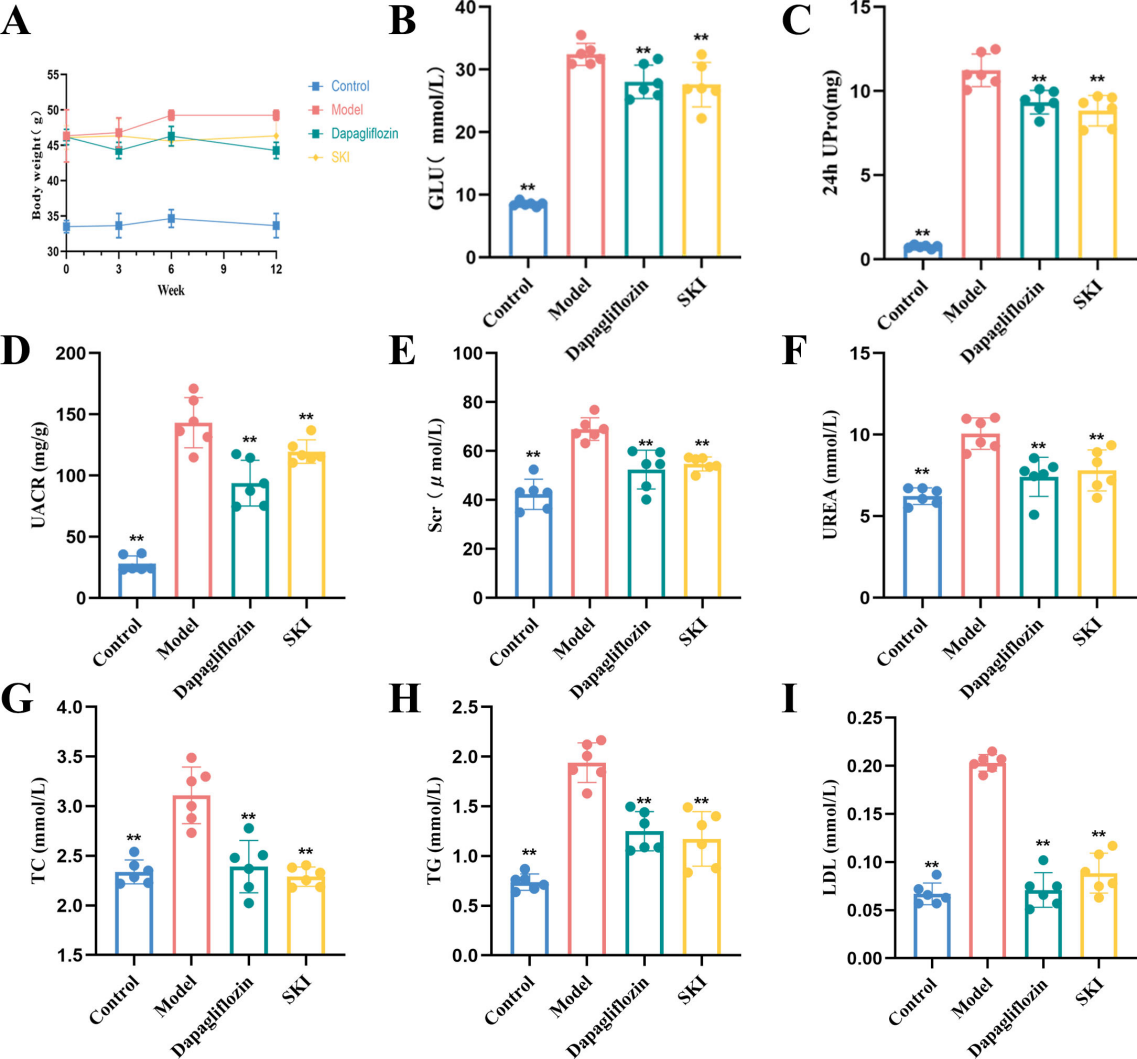
Effect of SKI on body weight **(A)**, blood glucose **(B)**, urinary protein **(C)**, UACR **(D)**, renal function **(E, F)**, and lipid profile **(G–I)** in db/db mice. *P <0.05, **P <0.01, compared with the model group. Data were analyzed by one way ANOVA (Mean ± SEM). N = 6. The x-axis represents different groups.

There was no significant difference in weight among the model, dagliflozin, and SKI groups before treatment (P>0.05) (**Figure 1A**). After treatment, the model group exhibited an increase in weight, while both the dagliflozin and SKI groups showed an overall downward trend, remaining lower than the model group. All three groups had consistently higher body mass than the normal group.

#### Effect of SKI on 24-h urine protein quantification and UACR in mice

After 12 weeks of treatment, 24-hour urinary protein quantification and UACR decreased significantly in both the dagliflozin and SKI groups compared to the model group (P<0.01, **Figures 1C, D**).

#### Effect of SKI on fasting blood glucose in mice

Compared with the model group, fasting glucose levels decreased significantly in both the dagliflozin and SKI groups compared to the model group (P<0.01, **Figure 1B**).

#### Effect of SKI on serum kidney function in mice

After 12 weeks of drug administration, SCr and urea levels increased significantly in the model group compared to the normal group (P<0.01, **Figures 1E, F**). The SKI group showed a significant decrease in these levels compared to the model group (P<0.01, **Figures 1E, F**).

#### Effect of SKI on serum lipids in mice

After 12 weeks of treatment, TC, TG, and LDL levels increased significantly (P<0.01, **Figures 1G–I**), while both the dagliflozin and SKI groups experienced a significant decrease compared to the normal group (P<0.05, P<0.01, **Figures 1G–I**).

#### Effect of SKI on renal histopathology in mice

**Figure 2:**
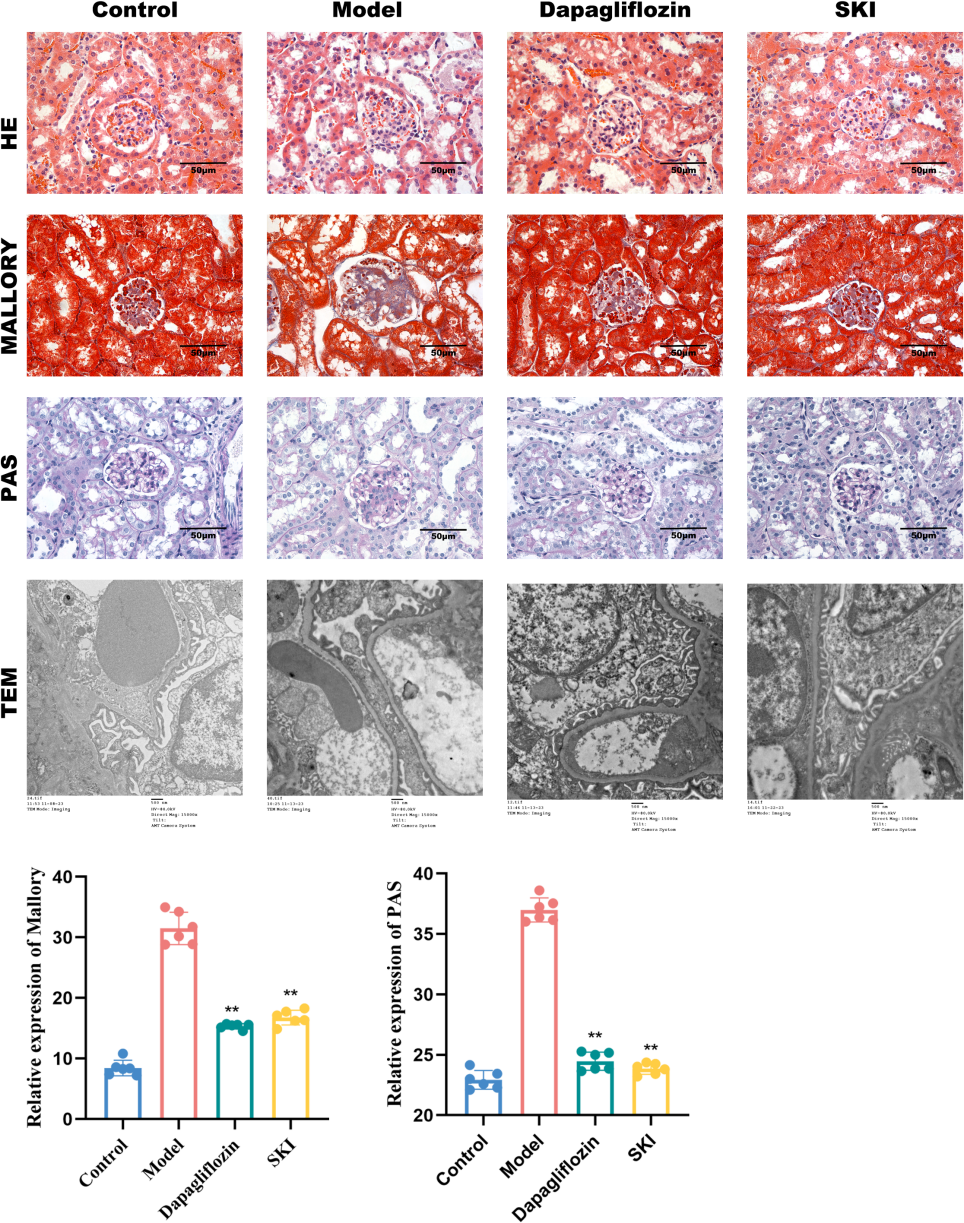
Kidney histopathology in DKD mice. HE (400X), Mallory (400X), Periodic acid-Schiff (PAS) (400X), TEM (1200X). **P <0.01, compared with the model group. The x-axis represents different groups.

HE staining revealed normal glomerular, tubular, and interstitial structures in the normal group. In contrast, the model group exhibited inflammatory cell infiltration, tubular epithelial cell damage, and moderate hyperplasia of glomerular mesangial cells. Compared to the model group, the SKI group showed decreased inflammatory cell infiltration and reduced mesangial cell hyperplasia. Mallory staining revealed a normal mesangial area and tubular interstitium in the normal group, while the model group exhibited severe collagen fiber hyperplasia in the mesangial region and tubular interstitium, with kidney fibrosis present in both the SKI and dagliflozin groups. Glycogen staining indicated that the mesangial matrix in the model group was significantly improved in the SKI and dagliflozin groups (**Figure 2**).

*TEM.* TEM observations of mouse glomerular foot processes revealed normal ultrastructure in the normal mice. The model group showed severe fusion of glomerular foot processes and pathological thickening of basement membrane segments. The basement membrane thickness was reduced in both the dagliflozin and SKI groups (**Figure 2**).

#### Effect of SKI on the expression of MCP-1 and CCR2 protein and mRNA in kidney tissues

**Figure 3:**
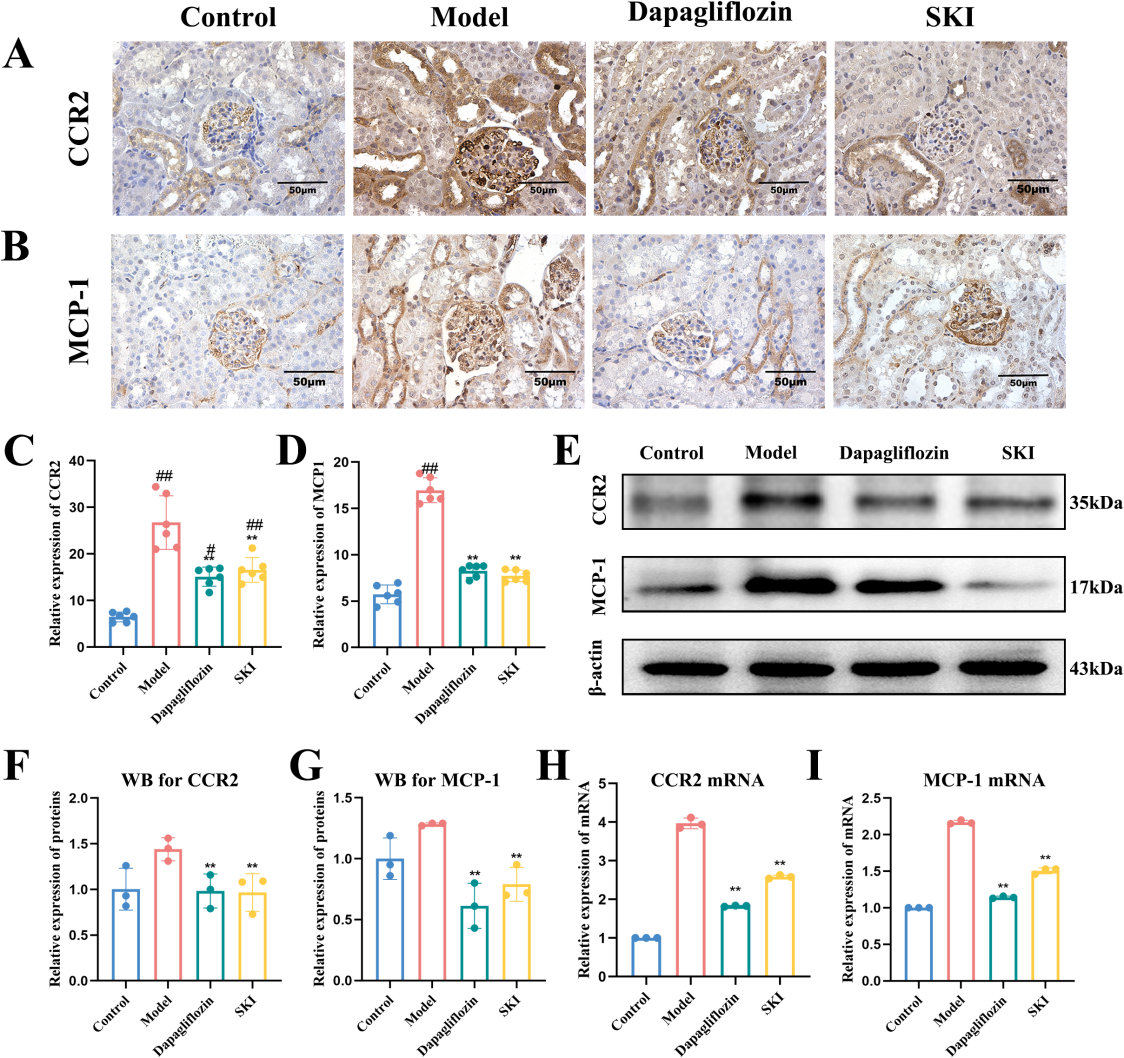
Protein and mRNA expression of CCR2 and MCP-1 in kidney tissues of mice in each group. **(A–D)** Expression and quantification of CCR2 and MCP-1 IHC. **(E)** WB expression of CCR2 and MCP-1. **(F)** Quantification of WB results for CCR2. **(G)** Quantification of WB results for MCP-1. **(H)** Quantification of CCR2 mRNA expression. **(I)** Quantification of MCP-1 mRNA expression. **P <0.01, compared with the model group. ^#^P <0.05, ^##^P <0.01, compared with the control group. Data were analyzed by one way ANOVA (Mean ± SEM). IHC: N = 6. WB and RT-PCR: N=3. The x-axis represents different groups.

Immunohistochemistry showed that the protein expression levels of MCP-1 and CCR2 increased in the glomeruli and tubular cytoplasm of the model group compared with the normal group (P<0.01, **Figures 3A–D**). In comparison to the model group, the expression of MCP-1 and CCR2 was significantly decreased in the renal tissues of db/db mice in the dagliflozin and SKI groups (P<0.01, **Figures 3A–D**). WB analysis revealed that MCP-1 and CCR2 protein expression increased in the model group compared with the normal group (P<0.01). Compared with the model group, MCP-1 and CCR2 expressions were significantly decreased in the dagliflozin and SKI groups (P<0.05, **Figures 3E–G**). Real-time qPCR demonstrated that MCP-1 and CCR2 mRNA expression increased significantly in the renal tissues of the model group compared with the normal group (P<0.01, **Figures 3H, I**). In contrast, MCP-1 and CCR2 mRNA expression in the dagliflozin and SKI groups decreased significantly compared to the model group (P<0.01, **Figures 3H, I**).

#### Effect of SKI on the protein expression of p38 and NFκB in kidney tissues

**Figure 4:**
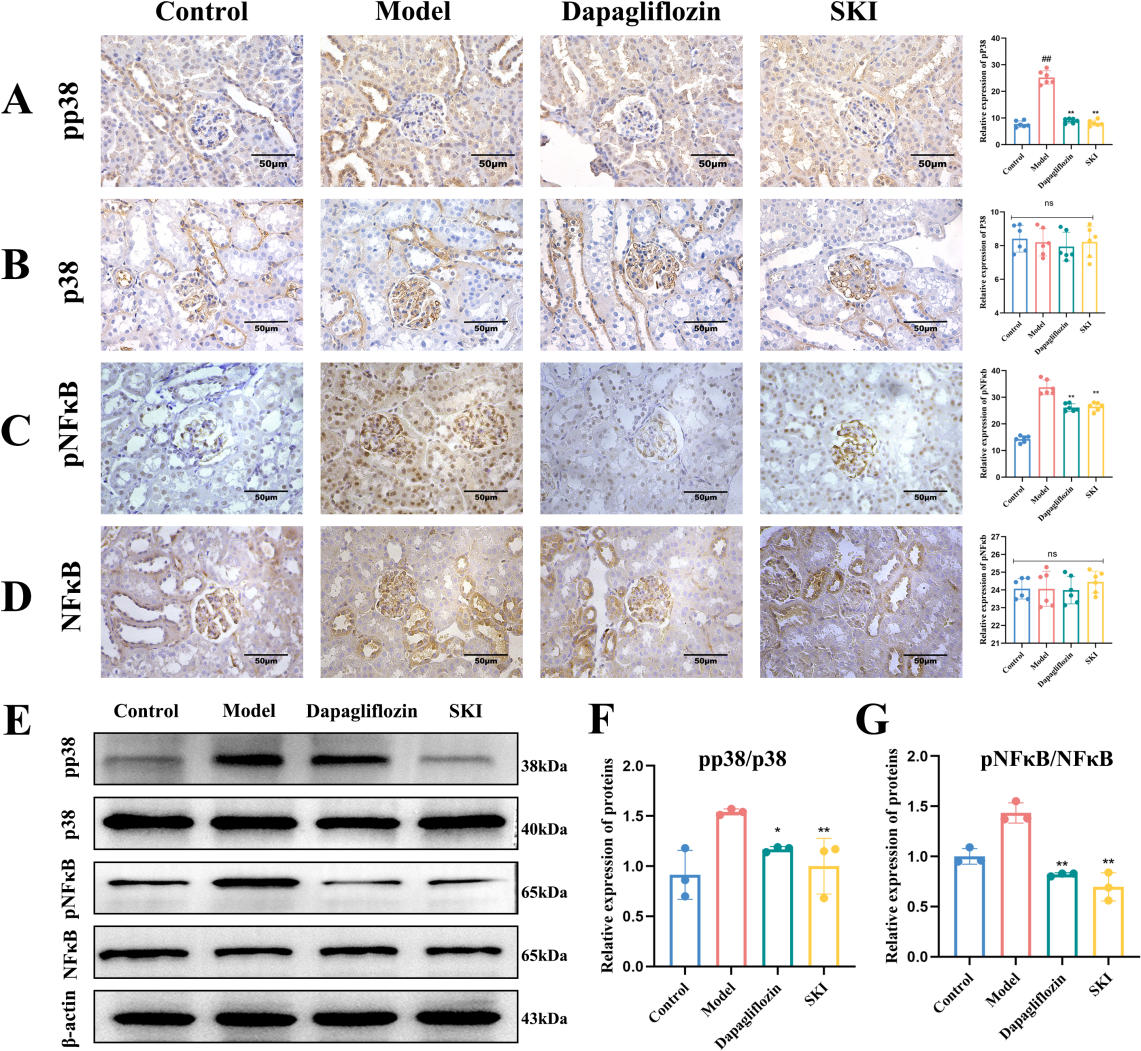
Protein expression of pp38 and p38 in kidney tissues of mice in each group. **(A–D)** Expression and quantification of p38, p-p38, p-NFκB, and NFκB by immunohistochemistry. **(E)** Western blot (WB) expression of p-p38, p38, p-NFκB, and NFκB. **(F)** WB quantification of p-p38/p38. **(G)** WB quantification of p-NFκB/NFκB. Data are compared with the normal group. *P <0.05, **P <0.01, compared with the model group. Data were analyzed by one way ANOVA (Mean ± SEM). IHC: N = 6. WB: N=3. The x-axis represents different groups.

Immunohistochemistry showed that p-p38 and p-NFκB protein expression increased in the glomeruli and tubular cytoplasm and nucleus compared with the normal group. Additionally, p-p38 and p-NFκB protein expression decreased in the dagliflozin and SKI groups (P<0.01, **Figures 4A–D**). There were no significant differences in p38 and NFκB protein expression between the groups. Furthermore, WB analysis indicated that the ratios of p-p38/p38 and p-NFκB/NFκB increased in the model group compared with the normal group (P<0.01, **Figures 4E–G**). In comparison to the model group, the expression of p-p38/p38 and p-NFκB/NFκB was significantly reduced in the dagliflozin and SKI groups (P<0.05, **Figures 4E–G**).

### Cell experiments

#### Effect of SKI on the activity of HK-2 cells

**Figure 5:**
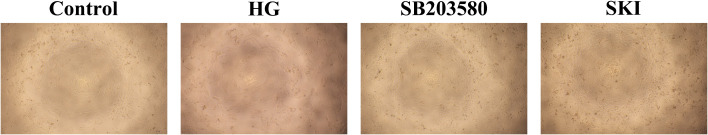
Cellular state 60 hours after drug treatment.

**Figure 6:**
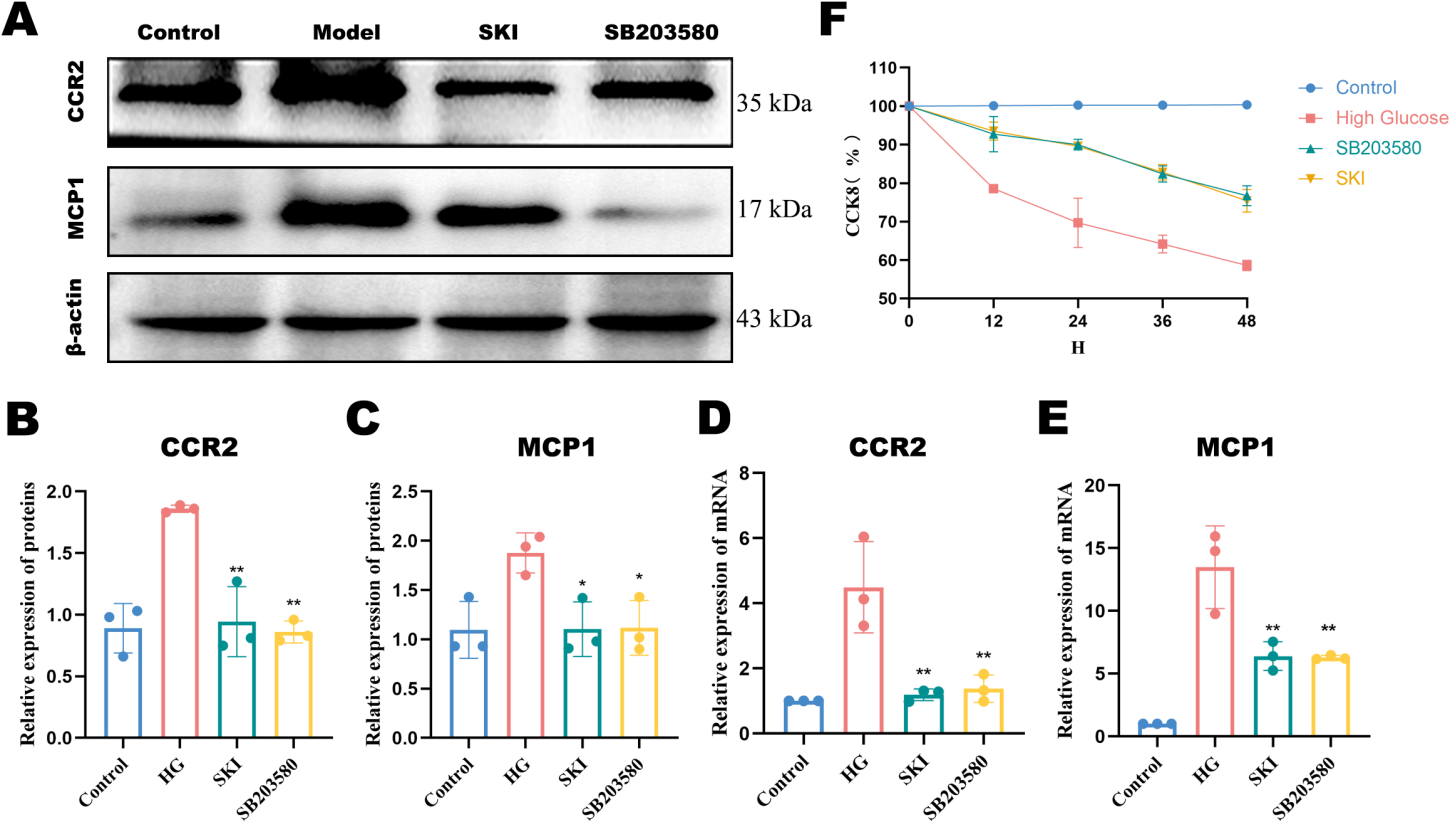
Cell viability and protein expression of CCR2 and MCP-1 in each group. **(A)** Histone expression as a loading control. **(B)** Western blot (WB) quantification of CCR2 protein expression. **(C)** WB quantification of MCP-1 protein expression. **(D)** mRNA expression levels of CCR2. **(E)** mRNA expression levels of MCP-1. **(F)** Cell viability in each group. *P <0.05, **P <0.01, compared with the model group. Data were analyzed by one way ANOVA (Mean ± SEM). CCK8: N = 6. WB: N=3. The x-axis represents different groups.

At 60 hours post-drug administration, the high-glucose group exhibited significant cellular damage compared to the other groups (**Figure 5**). The CCK-8 results showed that as time increased, the cell survival rate of each group gradually decreased compared with the normal group. The effect of high glucose on the survival rate became more apparent; however, the survival rate of the SB203580 and SKI groups increased compared with the high glucose group (**Figure 6F**).

#### Effect of SKI on MCP-1 and CCR2 protein and mRNA expression in HK-2 cells

WB analysis showed that p-p38/p38, MCP-1, and CCR2 were significantly increased in the high glucose group compared with the normal group (P<0.01, **Figures 6A–C**). Compared with the high glucose group, the expression of p-p38/p38, MCP-1, and CCR2 was significantly reduced in the inhibitor and SKI groups (P<0.05, **Figures 6A–C**). Real-time qPCR showed that MCP-1 and CCR2 mRNA expression levels were significantly increased in the high glucose group compared with the blank group (P<0.01, **Figures 6D, E**). Compared with the high glucose group, MCP-1 and CCR2 mRNA expression levels in HK-2 cells from the inhibitor SB203580 group and the SKI group were significantly reduced (P<0.01, **Figures 6D, E**).

#### Effect of SKI on the protein expression of p38 and NFκB in HK-2 cells.

**Figure 7:**
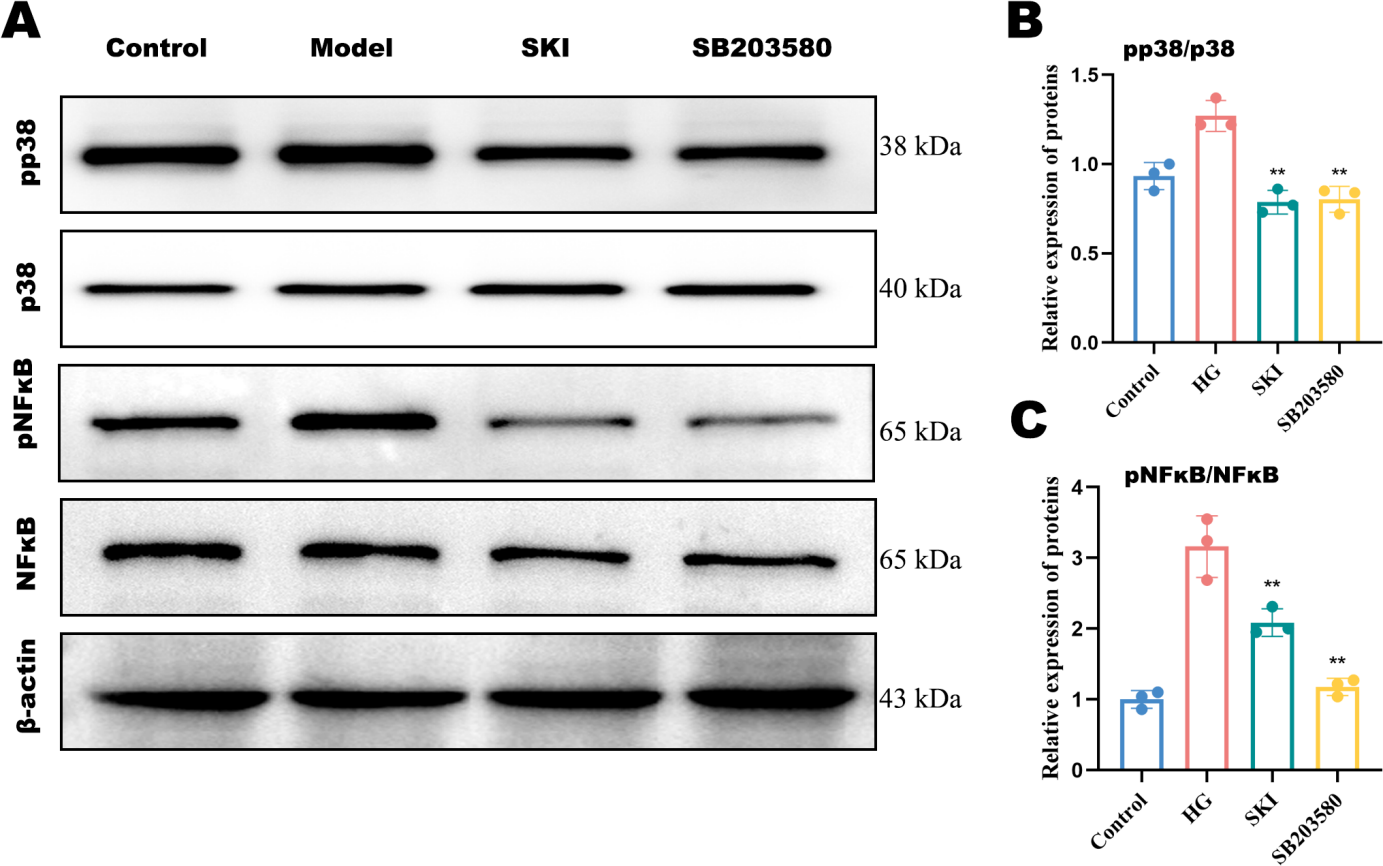
Protein expression of pp38 and p38 in HK-2 cells of each group. **(A–C)** Protein quantification of p-p38/p38 and p-NFκB/NFκB. *P <0.05, **P <0.01, compared with the model group. The x-axis represents different groups.

WB analysis showed that the protein expression of p-p38/p38 and p-NFκB/NFκB was significantly increased in the high glucose group compared with the normal group (P<0.01, **Figures 7A–C**). Compared with the high glucose group, the expression of p-p38/p38 and p-NFκB/NFκB was significantly reduced in the inhibitor and SKI groups (P<0.05, **Figures 7A–C**).

## Discussion

The pathological mechanisms of diabetic nephropathy primarily involve an impaired glomerular filtration barrier, glomerular mesangial expansion, and oxidative stress (10, 28). Hyperglycemia triggers cell damage, leading to the production of various pro-inflammatory mediators, such as interleukin 1 (IL-1) and tumor necrosis factor-α (TNF-α) (29). These pro-inflammatory factors contribute to the aggregation of macrophages and monocytes within the glomerulus. The accumulation of macrophages, in turn, generates cytokines, reactive oxygen species, and proteases, which drive fibrosis in the glomerulus. Furthermore, MCP-1 promotes the infiltration of macrophages (11). Importantly, studies have shown that reducing MCP-1 levels in mouse models of type 1 and type 2 diabetes mellitus can inhibit macrophage infiltration, thereby alleviating proteinuria and inflammatory markers (30). Studies have demonstrated that NF-κB p65 is a potential therapeutic target for DKD. Wogonoside, a bioactive compound derived from the traditional Chinese herb Astragalus membranaceus, ameliorates oxidative stress and inflammation in DKD by modulating multiple signaling pathways, thereby protecting podocytes and renal tubular epithelial cells (31, 32). These findings demonstrate that traditional Chinese medicine exhibits significant therapeutic potential and research value in the DKD.

Transitioning to therapeutic approaches, a meta-analysis investigating SKI combined with alprostadil for chronic kidney disease demonstrated a significantly higher response rate in the treatment group compared to the control group. Improvements were observed in creatinine clearance, urea nitrogen levels, and urinary protein quantification (33). Clinically, SKI is widely used for treating chronic kidney disease and has shown promise in managing diabetic nephropathy. Supporting this, animal studies on SKI injection revealed its ability to reduce diabetic tubular lesions by inhibiting epithelial-mesenchymal transition and endoplasmic reticulum stress, thereby achieving therapeutic effects (34). Further analysis of SKI injection’s composition identified active components such as oxycresol A, astragaloside IV, rhein acid, tanshinone IIA, and emodin (35). Studies on these components indicate that they collectively reduce inflammation in animal models (36–40). Specifically, emodin has been shown to mitigate the proliferation and infiltration of mesangial cells induced by high glucose (41).

Focusing on the structural integrity of the glomerulus, podocytes play a critical role as an essential barrier to glomerular filtration. Damage to podocytes can disrupt this barrier, leading to abnormal filtration rates (42). Consequently, proteinuria, often a consequence of podocyte injury, is an early and typical sign of kidney disease. In glomerulonephritis, MCP-1 and its receptor CCR2 are implicated in activating intracellular signaling pathways that regulate podocyte and HK-2 function (43, 44). Additionally, renal tubular epithelial vacuolar-like changes and fibrosis are pivotal in the progression of DKD (45). Our experimental findings align with these mechanisms. Mice in the model group exhibited significantly elevated 24-hour urinary protein levels and UACR compared to the normal group. Similarly, Scr and urea nitrogen levels were markedly increased, indicating substantial kidney injury. Pathological staining and electron microscopy further corroborated these findings, revealing severe glomerular mesangial cell hyperplasia, inflammatory cell infiltration, foot process fusion, and basement membrane thickening. Moreover, HE and Mallory staining highlighted mesangial cell proliferation and extensive collagen fiber deposition, as confirmed by Periodic acid-Schiff staining.

Following treatment with SKI injection, notable improvements were observed. Inflammatory cell infiltration was reduced, serum creatinine and urea nitrogen levels decreased, and glomerulosclerosis and interstitial fibrosis were alleviated (13, 35). Our experiments also demonstrated that SKI significantly decreased 24-hour urinary protein levels, Scr, and Urea in db/db mice. Furthermore, pathological staining showed that mesangial cell hyperplasia and inflammatory infiltration were substantially reduced in the SKI-treated group, with only minimal collagen fiber deposition observed with Periodic acid-Schiff staining. In parallel, electron microscopy showed improvements in podocyte structure, including reduced foot process fusion and thinner basement membranes in SKI-treated mice compared to the model group. In addition to these renal protective effects, literature and prior studies from our lab indicate that SKI exhibits hypoglycemic properties (14). SKI also appears to improve blood lipid profiles in DKD rats (46). Our current experiments confirmed that db/db mice treated with SKI exhibited reduced blood glucose levels, as well as significant improvements in total cholesterol, triglycerides, and LDL cholesterol.

Monocyte chemokines (such as MCP-1) play a crucial role in rapidly recruiting monocytes to sites of inflammation and tumors. Additionally, they promote macrophage infiltration in the kidney, making MCP-1 a key factor in the inflammatory response (11, 47). Studies using mouse models of type 1 and type 2 diabetes mellitus have demonstrated that the loss of MCP-1 reduces proteinuria and inflammation levels (30). Notably, while MCP-1 binds to multiple receptors, its preferred receptor is CCR2. Thus, the interaction between MCP-1 and CCR2 is vital for inflammation and inflammation-related diseases, as their combination facilitates the innate immune response by recruiting monocytes to inflammation sites (48).

Further insights reveal that the p38 MAPK signaling pathway significantly influences MCP-1 expression, which can alleviate inflammation and mitigate pain associated with inflammatory processes (49, 50). Inhibition of p38 MAPK phosphorylation has been shown to significantly suppress MCP-1 expression (51). Moreover, MCP-1 recruitment and production are closely linked to the NFκB signaling pathway (52). Typically, NFκB binds with its inhibitor IκB. Upon activation, IκBα is phosphorylated by IκB kinase, leading to its dissociation. This enables phosphorylated NFκB p65 to translocate to the nucleus, where it mediates the expression of inflammation-related genes. Consequently, this cascade promotes the expression of downstream target genes and proteins, triggering pathological states such as inflammation and immune responses (53–55). Supporting this, studies have shown that blocking the MAPK and NFκB pathways in inflammatory cells significantly reduces the expression of chemokines and pro-inflammatory cytokines. Specifically, inhibition of p38 and NFκB phosphorylation can suppress MCP-1 expression (56).

**Figure 8:**
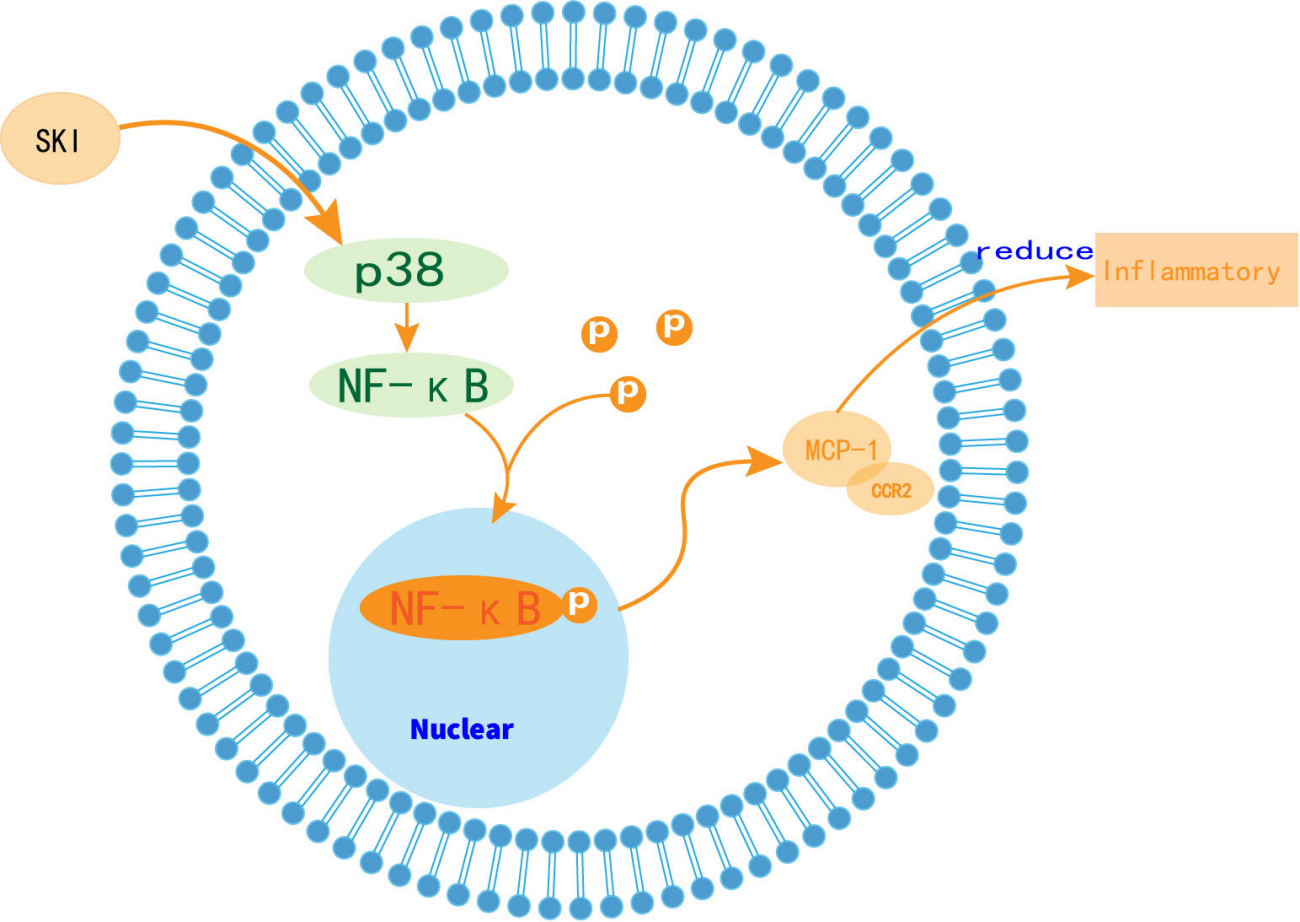
Schematic model of the effect of SKI on p38 MAPK/NF-κB/MCP1/CCR2 signaling pathway.

In our animal experiments, WB and RT-qPCR analyses revealed that the pro-inflammatory cytokine MCP-1 and its receptor CCR2 were significantly reduced in the model group. Correspondingly, phosphorylated p38 and NFκB levels were also markedly inhibited, indicating that SKI effectively alleviated the inflammatory response in the kidneys of db/db mice. These results were further corroborated by immunohistochemistry images, which provided a more intuitive visualization. In the model group, p-p38, pNFκB, MCP-1, and CCR2 exhibited substantial aggregation of buffy particles across the visual field. In contrast, the SKI-treated group displayed a significant reduction in these particles, with only small clusters located within glomeruli and renal tubules. This suggests that SKI can markedly improve kidney injury in diabetic nephropathy. Moreover, in experiments using high glucose-induced cells, the application of SB203580 (a p38 MAPK inhibitor) provided additional insights. Cell survival, as measured by CCK-8 assays, was notably lower in the high glucose-stimulated model group compared to the normal group. However, HK2 cells treated with the inhibitor and SKI showed improved survival under the same conditions. Protein content analyses of phosphorylated p38 and NFκB further supported these findings, as their expression levels were significantly reduced in the SKI group compared to the model group. Similarly, measurements of MCP-1 and CCR2 expression at both protein and mRNA levels indicated synchronous reductions. These results strongly suggest that SKI can inhibit MCP-1 and CCR2 via the p38 MAPK and NFκB signaling pathways, thereby alleviating inflammation and mitigating kidney damage in diabetic nephropathy (**Figure 8**).

## Conclusion

In conclusion, SKI demonstrates a protective effect in db/db mice by improving overall health, reducing 24-hour urinary protein levels, protecting kidney tissue, alleviating inflammation, and mitigating kidney injury. The underlying molecular mechanism likely involves the inhibition of MCP-1 and CCR2 expression through the suppression of p38 MAPK and NFκB signaling pathways, leading to a reduction in the inflammatory response.

## Data Availability

The datasets presented in this study can be found in online repositories. The names of the repository/repositories and accession number(s) can be found in the article/supplementary material.
